# Comparison between 1-year outcomes of aflibercept with and without photodynamic therapy for polypoidal choroidal vasculopathy: Retrospective observation study

**DOI:** 10.1371/journal.pone.0176100

**Published:** 2017-05-03

**Authors:** Kei Takayama, Hiroki Kaneko, Keiko Kataoka, Kyoko Hattori, Eimei Ra, Taichi Tsunekawa, Hiroshi Fukukita, Fuminori Haga, Yasuki Ito, Hiroko Terasaki

**Affiliations:** 1 Department of Ophthalmology, Nagoya University Graduate School of Medicine, Nagoya, Japan; 2 Department of Ophthalmology, National Defense Medical College, Tokorozawa, Japan; Massachusetts General Hospital, UNITED STATES

## Abstract

Polypoidal choroidal vasculopathy (PCV) is characterized by polyp-like choroidal neovascularization and a branching vascular network. Intravitreal aflibercept injection (IAI) or photodynamic therapy (PDT) is used for treatment. We retrospectively compared the 1-year outcomes of IAI monotherapy and its combination with initial PDT for PCV. Twelve eyes with naïve PCV received three IAIs and a single PDT after the first IAI and as needed injection (combination group); 11 eyes with naïve PCV received three IAIs and as needed injections (IAI group). Significant improvements in visual acuity after 2 months and in CRT after 1 month were maintained at 12 months in both groups (both *P* < 0.05); groups did not differ significantly at any time point. CCT significantly reduced after 3 and 12 months in the combination group (both *P* < 0.05) but not in the IAI group. A mean of 3.7 ± 0.9 and 5.6 ± 2.0 injections was administered to the combination and IAI groups, respectively (*P* = 0.013). Within a 1-year period, combination therapy was found to yield similar visual acuity and retinal structure improvements and maintenance as IAI monotherapy while requiring fewer IAIs.

## Introduction

Polypoidal choroidal vasculopathy (PCV) is characterized by polyp-like choroidal neovascularization and a branching vascular network as well as the presence of hyper-fluorescent nodules detectable during early-phase indocyanine green angiography [1, 2]. These characteristics are associated with serous exudation and subretinal hemorrhage, which lead to retinal pigment epithelium detachment and, occasionally, neurosensory retinal detachment [3–5]. The natural course of PCV may vary from relatively stable to repeated bleeding and leakage with vision loss and chorioretinal atrophy, with or without fibrotic scarring [6, 7]. Previous studies have demonstrated the efficacy of photodynamic therapy (PDT) and intravitreal anti-vascular endothelial growth factor (anti-VEGF) injection for the treatment of eyes with PCV [8–12]; in particular, combination therapy can exert synergistic effects on regressing polyps and better maintain visual acuity and retinal morphology when compared with PDT or anti-VEGF monotherapy. In addition, the great advantage of triple combination therapy composed of anti-VEGF injection, PDT, and subtenon steroid injection had been reported [13, 14]. Therefore, combination therapy should be considered as the first-line treatment for eyes with PCV [15, 16].

Aflibercept, a recombinant fusion protein, binds to and inhibits VEGF-A, VEGF-B, and placental growth factor [17]. This drug has been recently developed to afford a more potent and prolonged anti-VEGF effect than previous anti-VEGF drugs ranibizumab or bevacizumab. Intravitreal aflibercept injection (IAI) improves both visual acuity and retinal morphology in eyes with PCV, similar to other anti-VEGFs [18–20]. Recently, combination therapy of IAIs and PDT or triple therapy of IAI [21–23], PDT, and dexamethasone [24] have been reported to have preferable outcomes; however, to the best of our knowledge, studies which compare the outcomes of this combination therapy with or without PDT have not been previously reported. Therefore, the objective of this study was to compare the 1-year outcomes of IAI treatment with or without initial PDT for PCV.

## Methods

### Patients

This retrospective chart review was conducted at Nagoya University Hospital in Nagoya, Japan. Consecutive patients who visited the hospital with naïve subfoveal PCV between December 2012 and December 2014 and were able to complete follow-up visits arranged at 1-month intervals for a 12-month observation period were enrolled in the study. A clinical diagnosis of PCV was based on funduscopic examination findings of subretinal reddish-orange spheroidal lesions, and indocyanine green angiography findings of polypoidal vascular lesions, including branching vascular networks [1, 2, 5]. Patients who had eyes with wet AMD with PCV, previously undergone vitrectomy, laser photocoagulation, intravitreal triamcinolone injection, any intravitreal anti-VEGF injection, or PDT were excluded from the study.

Consecutive patients, who visited the hospital from December 2013 to December 2014, received three initial monthly IAIs and a single PDT session 3 days after the first IAI, followed by as needed treatment regimen (Combination group). Consecutive patients, who visited the hospital from December 2012 to December 2013, also received three initial monthly IAIs, followed by as needed treatment regimen (IAI group). During the monthly follow-up visit, vision test, IOP measurement, optical coherence tomography (OCT) were performed. Fluorescein angiography (FA) and indocyanine green angiography (ICGA) were performed for subjects requiring retreatment if exudative changes were observed on OCT.

Retrospective procedures conformed to the tenets of the World Medical Association's Declaration of Helsinki. The Nagoya University Hospital Ethics Review Board approved this retrospective analysis of patient data. Written informed consent was obtained from all patients prior to use their medical record data in the research.

### IAI injections

For each patient, a 0.12-mL aliquot of aflibercept (40 mg/mL) was prepared aseptically in a 1.0-mL syringe with a 30-gauge needle by the pharmaceutical department of our university hospital. After preparing the eye in a standard manner, a 0.05-mL volume (2.0 mg of aflibercept) was injected through the pars plana into the vitreous cavity 3.0–3.5 mm posterior to the limbus. After IAI, patients were instructed to apply topical antibiotics for 3 days. Patients received two additional IAIs per month, regardless of clinical findings. Thereafter, both groups were followed in accordance with the retreatment criteria of disease activity according to parameters recommended in the PRONTO study: evidence of persistent fiuid on OCT at least 1 month after the previous injection, an increase in OCT central retinal thickness (CRT) of at least 100 μmm, new macular hemorrhage, new area of classic CNV, or decrease in BCVA > 5 letters [25].

### PDT

PDT was administered to eyes in the combination group 3 days after the first IAI. A standard dose (6 mg/m^2^) of verteporfin was administered according to the protocol used in Treatment of Age-Related Macular Degeneration with Photodynamic Therapy studies [26, 27], as previously described [28–30]. The greatest linear dimension (GLD) and treatment spot size were measured using ICGA including PCV and branching vascular neovascularization, and the PDT treatment spot diameter was calculated as the GLD plus 1000 μm. A 689-nm laser system (Carl Zeiss, Dublin, CA, USA) was used to deliver 50 J/cm^2^ of energy to the treatment spot during an 83-second exposure.

### Best-corrected visual acuity

Best-corrected visual acuity (BCVA) was measured before IAI injection and 1 month after each IAI injection. A standard Japanese decimal visual acuity chart was used, and BCVA values were converted to Snellen chart and the Early Treatment Diabetic Retinopathy Study (ETDRS) letter chart similar to that followed in a previous study [31]. An increase of more than 15 letters was defined as a significant improvement, while a decrease of more than 15 letters was defined as significant deterioration.

### Central retinal thickness and central choroidal thickness measurements by OCT

Central retinal thickness (CRT) and central choroidal thickness (CCT) were measured using a Spectralis OCT^™^ system (Heidelberg, Dublin, CA) at baseline and monthly during the 12-month observation period. Six radial 6,000-μm-length scans were performed in enhanced depth imaging (EDI)-OCT mode. Each section was obtained using eye tracking, and an average of 100 B-scans was calculated. CRT was measured from the internal limiting membrane to Bruch’s membrane, and CCT was measured from the lower retinal pigment epithelium line to choroid-scleral junction using the caliper function of the machine. The measurements were conducted by two observers (K.T. and K.K.) and both observed were blinded to the patients’ clinical features. The average value of two investigators was used for the statistical analysis.

### Statistical analyses

The study outcomes included the BCVA, CRT, CCT, and percentage of eyes with fluid on OCT at the baseline and after 1, 2, 3, 6, 9, and 12 months; in addition, the numbers of IAIs administered during the 12-month period were compared between the groups. The Repeated measures ANOVA was used to compare the differences in the data within a group. The Mann–Whitney *U* test was used for intergroup data comparisons at the same time point. The Fisher exact test was used for the percentages of cases with fluid. A probability (P) value < 0.05 was considered statistically significant.

## Results

### Patient characteristics

The characteristics of the enrolled patients are listed in Table 1.

**Table 1:** Patient characteristics.

	Combination	IAI	*P* value*
**Number (eyes)**	12	11	
**Male/Female**	6/6	6/5	
**Mean age (years)**	72.9 ± 5.5	73.5 ± 4.9	0.50
**Mean visual acuity (letters score)**	69.1 ± 9.2	69.0 ± 13.2	0.30
**Mean central retinal thickness (μm)**	336 ± 63	328 ± 133	0.27
**Mean central choroidal thickness (μm)**	257 ± 68	222 ± 64	0.17
**Mean greatest linear dimension (μm)**	3487 ± 1203	3429 ± 1538	0.43
**Patterns of polyp (Solitary/Clustered)**	7/5	6/5	
**Mean number of polyps**	1.7 ± 0.9	1.5 ± 0.7	0.44
**Choroidal vascular hyperpermeability (eyes)**	3	1	

*: Mann–Whitney U test, Values are shown as means ± standard deviations.

Twelve patients (12 eyes; six male and six female patients, all Japanese, mean age = 72.9 ± 5.5 years) were included in the combination group, and 11 patients (11 eyes; six male and five female patients, all Japanese; mean age = 73.5 ± 4.9 years) were included in the IAI group. The baseline mean BCVAs were 69.1 ± 9.2 letters score in the combination group and 69.0 ± 13.2 letters score in the IAI group. The baseline mean CRTs were 336 ± 63 μm in the combination group and 328 ± 133 μm in the IAI group, and the baseline mean CCTs were 257 ± 68 μm in the combination group and 222 ± 64 μm in the IAI group. The mean GLDs were 3487 ± 1203 μm in the combination group and 3429 ± 1538 μm in the IAI group. In the combination group, 7 eyes had solitary polyp and 5 eyes had clustered polyp, and 3 eyes had choroidal vascular hyperpermeability. Mean number of polyps was 1.7 ± 0.9. In the IAI group, 6 eyes had solitary polyp and 5 eyes had clustered polyp, and 1 eye had choroidal vascular hyperpermeability. Mean number of polyps was 1.5 ± 0.7. No significant differences in any other parameters were observed between the groups.

### BCVA

**Fig 1:**
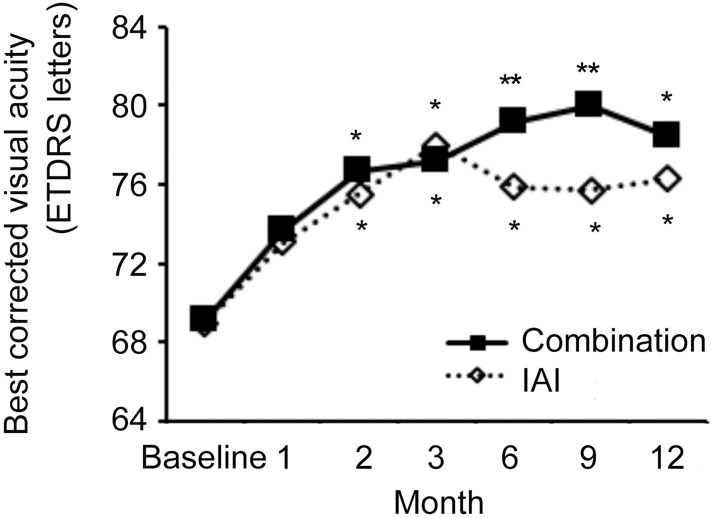
Best-corrected visual acuity (BCVA) outcomes in the two study groups. Improvements in BCVA were maintained at 12 months in both groups, and no significant differences were observed between the groups at any time point. * *P* < 0.05 by Repeated measures ANOVA.

The visual acuity outcomes are shown in Fig 1 and Table 2.

**Table 2:** Outcome of visual acuity.

	Baseline	1M	2M	3M	6M	9M	12M
Combination	69.1 ± 9.2	72.7 ± 11.3	76.7 ± 5.2*	77.2 ± 6.4*	79.2 ± 8.8**	80.0 ± 6.4**	78.1 ± 9.0*
IAI	69.0 ± 13.2	73.0 ± 13.0	75.5 ± 11.4*	76.5 ± 12.0*	76.0 ± 12.0*	75.0 ± 11.6*	75.9 ± 10.9*

Repeated measures ANOVA

*: P < 0.05

**: P < 0.01;

Values are shown as means ± standard deviations.

**Fig 2:**
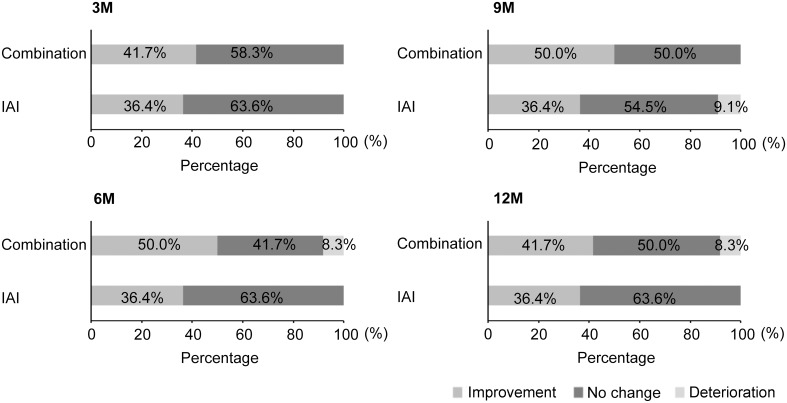
Changes in best-corrected visual acuity at 3, 6, 9, and 12 months in the two study groups. Visual acuity improvements were maintained at 12 months in both groups, and no significant inter-group differences were observed at any time point.

In the combination group, the baseline mean BCVA was 69.1 ± 9.2 letters score, with follow-up values of 72.7 ± 11.3, 76.7 ± 5.2, 77.2 ± 6.4, 79.2 ± 8.8, 80.0 ± 6.4, and 78.1 ± 9.0 letters score at 1, 2, 3, 6, 9, and 12 months, respectively, representing a significant improvement from the baseline beginning at 2 months (*P* < 0.05 at 2, 3, and 12 months and <0.01 at 6 and 9 months) In the IAI group, the baseline mean BCVA was 69.0 ± 13.2 letters score, followed by 73.0 ± 13.0, 75.5 ± 11.4, 76.5 ± 12.0, 76.0 ± 12.0, 75.0 ± 11.6, and 75.9 ± 10.9 letters score at 1, 2, 3, 6, 9, and 12 months, respectively, which also represented significant improvement from the baseline after 2 months (*P* < 0.05 at 2, 3, 6, 9, and 12 months) Changes in visual acuity at 3, 6, 9, and 12 months are shown in Fig 2.

### CRT and CCT

**Fig 3:**
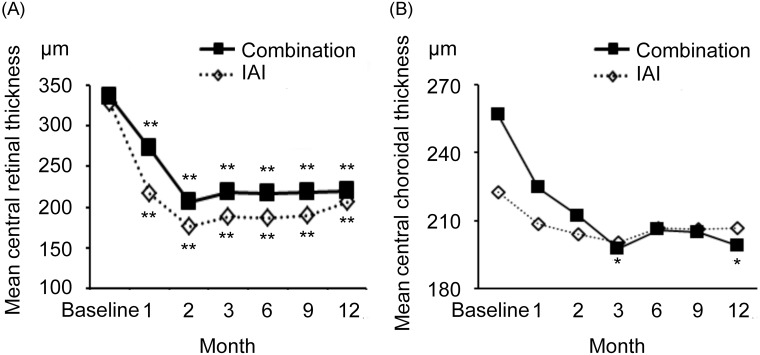
Central retinal thickness (CRT) and central choroidal thickness (CCT) outcomes in the two study groups. **(A)** Reductions in CRT were maintained at 12 months after both treatments, and no significant differences were observed between the groups at any time point during the 12-month study period. **(B)** A significant reduction in CCT from baseline to 3 and 12 months was observed in the combination group. No significant differences were observed between the two groups at any time point. * *P* < 0.05, ** *P* < 0.01 by Repeated measures ANOVA.

CRT and CCT outcomes are shown in Fig 3 and Table 3.

**Table 3:** Outcomes of central retinal thickness and central choroidal thickness.

	Baseline	1M	2M	3M	6M	9M	12M
**Central retinal thickness (μm)**							
Combination	336 ± 63	272 ± 73**	207 ± 45**	218 ± 48**	217 ± 42**	219 ± 50**	219 ± 54**
IAI	328 ± 133	217 ± 78**	175 ± 34**	188 ± 31**	188 ± 44**	189 ± 50**	206 ± 55**
**Central choroidal thickness (μm)**							
Combination	257 ± 68	224 ± 64	212 ± 62	198 ± 63*	206 ± 59	205 ± 59	199 ± 46*
IAI	222 ± 64	208 ± 58	204 ± 49	200 ± 49	207 ± 56	206 ± 59	207 ± 57

Repeated Measures ANOVA

*: P < 0.05

**: P < 0.01,

Values are shown as means ± standard deviations.

In the combination group, the baseline mean CRT was 336 ± 63 μm; this value decreased significantly to 272 ± 73 μm, 207 ± 45 μm, 218 ± 48 μm, 217 ± 42 μm, 219 ± 50 μm, and 219 ± 54 μm at 1, 2, 3, 6, 9, and 12 months, respectively (*P* < 0.01 at all-time points beyond the baseline). In the IAI group, the baseline mean CRT was 328 ± 133 μm; this value decreased significantly to 217 ± 78 μm, 175 ± 34 μm, 188 ± 31 μm, 188 ± 44 μm, 189 ± 50 μm, and 206 ± 55 μm at 1, 2, 3, 6, 9, and 12 months, respectively (*P* < 0.01 at all-time points beyond the baseline). In summary, CRT reductions were maintained at 12 months in both groups, and there were no significant intergroup differences during the observation period.

In the combination group, the baseline mean CCT was 257 ± 68 μm, followed by 224 ± 64 μm, 212 ± 62 μm, 209 ± 63 μm, 220 ± 60 μm, 225 ± 59 μm, and 214 ± 50 μm at 1, 2, 3, 6, 9, and 12 months, respectively. A significant reduction relative to the baseline CCT was observed at 3 and 12 months (both *P* < 0.05). In the IAI group, the baseline mean CCT was 222 ± 64 μm, followed by 208 ± 58 μm, 204 ± 49 μm, 200 ± 49 μm, 207 ± 56 μm, 206 ± 59 μm, and 207 ± 57 μm at 12 months, respectively. No significant intergroup differences were observed at any time point.

### Numbers of IAI

**Fig 4:**
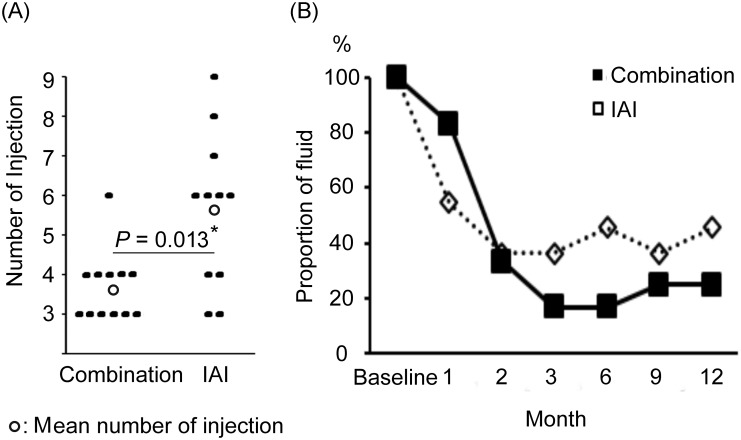
Numbers of intravitreal aflibercept injections (IAI) administered to the two study groups. **(A)** Significantly fewer IAIs were administered to the combination group than to the IAI group. **(B)** The groups had no significant difference in terms of the percentage of cases over 12 months. * *P* < 0.05 by Mann–Whitney *U* test.

The mean numbers of IAIs administered in the two groups are shown in Fig 4A.

After 3-monthly consecutive injections, four, one, and seven cases in the Combination group required one, three, and no additional injections, respectively, for an average of 3.7 ± 0.9 injections during the 12-month study period. In the IAI group, two, three, one, two, one, and two cases required one, three, four, five, six, and no injections, respectively, for an average of 5.6 ± 2.0 injections during the 12-month period. In summary, the combination group required significantly fewer IAIs than did the IAI group (*P* = 0.013).

The percentages of cases with persistent or recurrent subretinal or intraretinal fluid are shown in Fig 4B. In the combination group, this value decreased by 83.3%, 33.3%, 16.7%, 16.7%, 25%, and 25% at 1, 2, 3, 6, 9, and 12 months, respectively. In the IAI group, decreases of 54.5%, 36.4%, 36.4%, 45.5%, 36.4%, and 45.5% were observed at 1, 2, 3, 6, 9, and 12 months, respectively. The groups had no significant difference in terms of the percentages of cases with fluid at any month.

## Discussion

This study compared outcomes of visual acuity, CRT, CCT, number of IAIs, and frequency of retinal fluid among cases of eyes with PCV in the Combination and IAI groups during a 12-month study period. Notably, combination therapy was found to yield similar visual acuity and retinal structure improvements and maintenance as IAI monotherapy while requiring fewer IAIs. Initial IAI with PDT have been reported to have preferable outcomes. Matumiya et al. [21] demonstrated outcomes of initial three IAI with PDT, and Kikushima et al. [23] demonstrated the comparison of initial three IAI versus one IAI with PDT. Ho et al. [24] reported triple therapy with IAI, PDT, and subtenon steroid injection. To the best of our knowledge, it is the first report of the comparison of three IAI versus three IAI with PDT. The outcome of visual and anatomical improvements over 12 month follow-up in the present study could be same as that observed in the study by Matsumiya [21].

Previous studies have reported the efficacy of PDT with other VEGF therapies for PCV and have compared combination therapies and monotherapies. For example, the EVEREST study prospectively compared the 6-month outcomes of anti-VEGF monotherapy and combination therapy and found that the latter was statistically superior in terms of achieving complete polyp regression and reducing CRT, with no significant differences in visual acuity [32]. During a 12-month retrospective observation in another study, both combination therapy and monotherapy were found to improve visual acuity and reduce CRT, with no significant intergroup differences; additionally, fewer injections were required in the combination group [33]. As in these previous studies, the current study observed similar outcomes and a lack of adverse events with combination therapy and IAI monotherapy.

PDT and anti-VEGF treatment lead to a decrease in CCT associated with PCV, and recurrence is characterized by the return of the post-treatment CCT to the baseline level [29]. Therefore, cChanges in choroidal thickness after treatment may reflect disease activity. In previous studies, PDT monotherapy and ranibizumab monotherapy reduced CCT by 85.1% and 95% from the baseline at 12 months [34]. Despite differences in the cases that preclude comparison, in the current study IAI monotherapy led to a non-significant decrease in the mean CCT from 222 μm at baseline to 207 μm at 12 months, whereas combination therapy led to a significant decrease from 257 μm at the baseline to 199 μm at 12 months. No significant intergroup differences in CCT were observed at any time points, although we compare the changes of CCT at 3 and 12 month between the two groups, and there was a significant difference between the two groups at 3 month and tendency to be different at 12 month (P = 0.035 and P = 0.08 by Mann–Whitney U test).

Recent studies of PCV treatment have reported a reduction in choroidal thickness after anti-VEGF injection [35, 36] and confirmed that at the time of PCV recurrence, the subfoveal choroidal thickness returned to the baseline level after thinning as a result of PDT [37, 38]. Although we could not compare our combination therapy with others, we found it better able to reduce CCT and disease activity than IAI monotherapy. Accordingly, the combination group would need fewer IAI injections than the IAI monotherapy group during the 12-month study period. For elderly population, lower frequency of IAI would be quite important because they do not travel well (especially in the case of low VA) and sometimes forget appointments, and possible adverse events.

Four limitations of this study that should be noted are the small number of patients, short observation period, single center study, and retrospective nature; therefore, no randomization was observed. A previous study of anti-VEGF with PDT also included a small case series and reported results similar to those of our study [8–12], and in the treatment of PCV, the long-term outcome of anti-VEGF therapy with PDT is less positive than that of anti-VEGF monotherapy [39]. Long-time observation would be needed.

However, another study that involves more cases and a longer observation period would be needed to better estimate the benefits of combination treatment.

## Conclusion

Our results demonstrate that for eyes with PCV, IAI with PDT and IAI monotherapy yielded similar improvements in visual acuity and retinal structure, and the former required fewer injections. We believe that our results demonstrate the efficacy of this combination therapy, although additional studies with longer observation periods are needed for confirmation.

## Supporting information

S1 Materials The date of patients included in the study. (XLSX)
